# Acute Acrolein Exposure Induces Impairment of Vocal Fold Epithelial Barrier Function

**DOI:** 10.1371/journal.pone.0163237

**Published:** 2016-09-19

**Authors:** Xinxin Liu, Wei Zheng, M. Preeti Sivasankar

**Affiliations:** 1 School of Health Sciences, Purdue University, West Lafayette, Indiana, United States of America; 2 Weldon School of Biomedical Engineering, Purdue University, West Lafayette, Indiana, United States of America; 3 Department of Speech, Language, and Hearing Sciences, Purdue University, West Lafayette, Indiana, United States of America; UNIVERSITY OF CENTRAL FLORIDA, UNITED STATES

## Abstract

Acrolein is a ubiquitous pollutant abundant in cigarette smoke, mobile exhaust, and industrial waste. There is limited literature on the effects of acrolein on vocal fold tissue, although there are clinical reports of voice changes after pollutant exposures. Vocal folds are responsible for voice production. The overall objective of this study was to investigate the effects of acrolein exposure on viable, excised vocal fold epithelial tissue and to characterize the mechanism underlying acrolein toxicity. Vocal fold epithelia were studied because they form the outermost layer of the vocal folds and are a primary recipient of inhaled pollutants. Porcine vocal fold epithelia were exposed to 0, 50, 100, 500, 900 or 1300 μM of acrolein for 3 hours; the metabolic activity, epithelial resistance, epithelial permeability, tight junction protein (occludin and claudin 3) expression, cell membrane integrity and lipid peroxidation were investigated. The data demonstrated that acrolein exposure at 500 μM significantly reduced vocal fold epithelial metabolic activity by 27.2% (p≤0.001). Incubation with 100 μM acrolein caused a marked increase in epithelial permeability by 130.5% (p<0.05) and a reduction in transepithelial electrical resistance (TEER) by 180.0% (p<0.001). While the expression of tight junctional protein did not change in acrolein-treated samples, the cell membrane integrity was significantly damaged with a 45.6% increase of lipid peroxidation as compared to controls (p<0.05). Taken together, these data provide evidence that acute acrolein exposure impairs vocal fold epithelial barrier integrity. Lipid peroxidation-induced cell membrane damage may play an important role in reducing the barrier function of the epithelium.

## Introduction

The vocal folds are paired, multi-layered, membranous tissues within the larynx. Vocal fold vibration is flow-induced and occurs between 100–300 times per second in conversational speech. Intact vocal fold abduction and adduction are also essential for respiration and healthy swallowing [1]. The outermost surface of vocal folds consists of 5 to 10 cell layers of squamous epithelial cells with tight junctions [2]. The epithelium forms a physical barrier to prevent inhaled xenobiotic penetration and protect underlying connective tissue and muscle. This stratified structure is unique compared to epithelia in other parts of respiratory system. Besides being a barrier, the vocal fold epithelium also secretes mucins, transports ions, and is associated with water fluxes to actively control surface composition [3–5].

The effects of exogenous insults, such as simulated gastric reflux, on the barrier function of vocal fold epithelium has been reported in the literature [6–8]. These noxious insults can compromise the epithelial barrier as measured by decreased epithelial resistance [8–10]. Tobacco smoke, for example, is an abundantly studied pollutant; 3-month exposure in rabbits causes hyperplasia with disturbed stratification on vocal fold epithelium [11]. A reduction in desmosomes and enlargement of intercellular space has been observed in rats following 60 day tobacco exposure [12].

Acrolein, an unsaturated aldehyde with a high electrophilicity, is one of the major toxicants present in cigarettes (about 10–500 μg/cigarette) [13]. It is also formed by the combustion of fossil fuels, woods, plastics, and heating of animal fat [14–17]. At room temperature, acrolein is present as a liquid, but is highly volatile. It also exists in the environment as a gas; this gas can contact the airway epithelium when inhaled. The current literature suggests that the mechanisms by which acrolein causes toxicity pertain to interaction with nucleophiles in a variety of local cellular structures [13, 18–21], induction of oxidative stress [21, 22] with ensuing lipid peroxidation, and covalent binding with proteins to form adducts. Studies also show that acrolein acts as a mutagen, leading to damaged DNA and inhibited DNA repair in lung cells [13]. Moreover, it interferes with the immune response in the respiratory tract [23–28]. Whether and how acrolein directly affects the apical vocal fold epithelia, the first line of defense to foreign insults, is not known.

Voice problems including hoarseness and lowered fundamental frequency are commonly seen among smokers [29]. Smoking is reportedly capable of increasing the permeability and damaging the cell membrane in type I pneumocyte in guinea pig [30]. The application of cigarette smoke condensate to ex vivo porcine tissue did not alter epithelial barrier function [31], but these negative findings could be attributed to the acute exposure duration and dosage selected for study. Another reason for the non-effect could be that the cigarette smoke condensate contains only smoke particulates. The effects of components such as acrolein, which are mainly contained in the gaseous phase were not examined. Acrolein is almost entirely found in the gaseous phase of mainstream smoke [32, 33] and may play a role in vocal fold damage. Subchronic exposure of rats to acrolein for 13 weeks induces inflammation and hyperplasia in the respiratory tract including the larynx [25]. Another acute study on vocal fold epithelium shows a reduction of sodium ion transport after 1 hour exposure with acrolein [3]. Nonetheless, the mechanism whereby acrolein affects the vocal fold epithelial barrier remains elusive.

This current study was designed to investigate the effects of acrolein on vocal fold epithelial barrier integrity. We hypothesized that acute exposure to acrolein may result in a dose-dependent reduction in vocal fold epithelial viability. We also hypothesized that acrolein exposure may impair epithelial barrier integrity as indicated by a decreased epithelial resistance and an increased epithelial permeability. We further hypothesized that the impairment of barrier integrity may be caused by altered tight junction protein expression and/or cell membrane damage. The findings from this work will provide the groundwork to understand the effects of acrolein on vocal fold epithelia pathophysiology and its health impact on the human voice.

## Materials and Methods

This study is exempt from the Institutional Animal Care and Use Committee (IACUC) of Purdue University, because the tissues were obtained from the slaughterhouse after sacrifice of pigs. Details are provided below.

### Materials

Protease Inhibitor Cocktail was purchased from Calbiochem (San Diego, CA); bovine serum albumin (BSA) standards and Pierce LDH cytotoxicity assay kit from Thermo Scientific (Rockford, IL); Tris, sodium dodecyl sulfate (SDS), cDNA synthesis kit, iTaq Universal SYBR Green Supermix, 2xLaemmli sample buffer and PVDF membrane from Bio-Rad (Hercules, CA). Primary rabbit anti-occludin antibody (ab31721) and primary mouse anti-4 HNE antibody (ab48506) were purchased from Abcam (Cambridge, MA); anti-rabbit IgG-HRP (goat) and anti-mouse IgG-HRP (goat) from Santa Cruz Biotechnology (sc-2004, sc-2005, Dallas, TX); enhanced chemiluminescene reagent (ECL) from Pierce Endogen (Rockford, IL); Alexa Fluor 488 goat anti-rabbit IgG (H+L) antibody (A-11034) from Thermo Scientific (Rockford, IL); and cyanine Cy^™^3 Goat Anti-Mouse IgG (H+L) (115-165-166) from Jackson ImmunoResearch Inc (West Grove, PA). Primers for qPCR analysis were obtained from Integrated DNA Technologies (Coralville, Iowa). Acrolein (99%), primary mouse anti-β-actin antibody and other chemicals were purchased from Sigma Aldrich (St Louis, Missouri). All reagents were of analytical grade, HPLC grade, or the best available pharmaceutical grade.

### Vocal fold preparation

Seventy-two fresh male and female porcine larynges were obtained from two local, Indiana state-inspected and approved abattoirs and transported in cold phosphate buffered saline (PBS) to the lab. The larynges were dissected following protocols utilized in previous published studies [3, 6]. In brief, the larynges were bisected along the midsagittal plane. The epithelium, basal lamina, and superficial lamina propria (referred as vocal fold epithelia below) were dissected from the larynges, and challenged with control (Hanks Balanced Salt Solution; HBSS), or acrolein in HBSS. The duration of challenge was 3 hours for all experiments. This duration was selected to represent an acute exposure model.

### Epithelial metabolic activity assay

Vocal fold epithelial metabolic activity was assessed using a modified MTT assay following a published protocol [34]. Epithelia samples were dissected from 7 larynges (fourteen vocal folds), punched (4 mm in diameter), and weighed. Samples were incubated in oxygenated (95% O_2_ and 5% CO_2_) medium for 3 hr at 37°C in 7 groups: positive control (tissue boiled), control (HBSS), 50 μM acrolein, 100 μM acrolein, 500 μM acrolein, 900 μM acrolein and 1300 μM acrolein. Boiled tissue were used as positive controls since they are non-viable. Samples were then incubated in the MTT solution with 100 rpm rotation for 2 hr and rinsed with PBS. Finally, an aliquot (4 mL) of DMSO was added to each well to extract formazan while the tissues were completely minced. The absorbance of formazan was detected using an ELISA scanner (SpectraMax M2e, Molecular Devices, Sunnyvale, CA) at 570 nm. The viability index was calculated as the ratio of absorbance to the tissue weight (abs/mg). The viability index was normalized by the mean of that in control group to the percentage. Since the MTT revealed reduction of metabolic activity at acrolein concentrations ≥ 500 μM, we chose 100 μM, which did not reduce the metabolic activity of the epithelial tissue, as the concentration of acrolein in the treated group in the ensuing experiments. This enabled us to avoid any interference from reduced metabolic activity in the experiments below.

### Determination of transepithelial electrical resistance (TEER)

An Ussing chamber system (model 15362, World Precision Instruments, WPI, Sarasota, FL) was used for assessment of TEER values. Fourteen vocal fold epithelia were mounted on Lucite chambers that were filled with oxygenated HBSS, warmed to 37°C. Tissues were maintained in their entirety as a single specimen as they were dissected, approximately 1.5 cm long, 1 cm wide and 1 mm thick. Epithelia with TEER values ≥ 300 Ω*cm^2^ were considered viable and were used for this study [35]. This threshold for vocal fold epithelial viability is based on published literature [3, 6, 8]. The apical side of the epithelia was exposed to either 100 μM acrolein or control (without acrolein). The TEER values were determined using a voltage clamp (model DVC-1000, WPI, Sarasota FL) and DataTrax (WPI, Sarasota, FL). These techniques are routinely used in studies of airway epithelial physiology [36, 37]. An instant 2mV potential from voltage clamping equipment was presented to the tissue every two minutes, and the instant change of current was recorded by the software. The changed current was normalized by the area of the chamber where the tissue contacts with the solution (1.13 cm^2^) and the resistance (TEER value) was calculated using the format R = V/I, where R stands for resistance, V represents potential and I means the normalized current. The change of TEER value were calculated using the TEER value prior to and following 3 hours after acrolein exposure. The change of the TEER value in the two groups were compared and percentage of change between the two groups were calculated. Seven vocal fold samples per group met the criteria for viability for a total of 14 vocal fold samples for this methodology.

### Assessment of epithelial permeability

Epithelial permeability was measured in epithelia mounted on Ussing chambers (above). Fourteen epithelial tissues were exposed to 100 μM acrolein or control without acrolein. The experiments were conducted at the same time as the TEER experiments described above. Immediately following the treatment, an aliquot of 1 mg/mL sodium fluorescein (NaFl), a permeability marker, was added to the apical chamber. Samples (200 μL) of the medium on the basolateral side of the tissues were collected prior to and following 3 hours of acrolein/control exposure. Fluorescence intensities were detected in duplicate using an ELISA scanner (SpectraMax M2e, Molecular Devices, Sunnyvale, CA) with the wavelength setting at 480 nm for excitation and 525 nm for emission. The permeability index represents the percentage of the fluorescein marker passing through the epithelial tissue. The permeability index was calculated by the change of the fluorescein intensity on the basolateral side divided by the fluorescein intensity on the apical side right after the sodium fluorescein was added and mixed.

### Determination of Levels of mRNAs encoding tight junctional proteins

The levels of mRNA encoding claudin3 and occludin were quantified using quantitative real-time polymerase chain reaction. Twelve vocal fold epithelial samples were incubated with or without 100 μM acrolein. The total RNA was isolated from the epithelia and purified using NucleoSpin RNA isolation & purification kit following the manufacturer’s direction. RNA was then reverse transcribed into cDNA using a Bio-rad iScript cDNA synthesis kit. The real-time PCR was conducted in the CFX Connect Real-Time PCR Detection system (Bio-Rad, CA). The qPCR was performed using a protocol with an initial 3-min denaturation at 95°C; then 40 cycles of 30-sec denaturation at 95°C, 10-sec gradient 55.0°C– 65.0°C and 30-sec extension at 72°C. Each of the qPCR samples were run in duplicate using the iTaq Universal SYBR Green Supermix (Bio-Rad) kit. The amplification efficiencies of target and reference genes were examined by the amplification of a series of dilutions of control templates. ΔΔCt, the threshold cycle time value, was used for the evaluation of relative mRNA expression of the target genes between control and acrolein treated groups. The target genes Ct values were normalized with that of β-actin in the same sample. The relative expressions of target genes expression in acrolein compared to control were calculated by setting the control as 100%.

The forward and reverse primers for Claudin3 and Occludin genes were designed using Primer Express 3.0 software. The primer sequences for porcine claudin3 used in this study were: forward primer 5-CCTACGACCGCAAGGACTAC -3 and reverse primer 5-CATCTGGGTGGACTGGTCTC-3 (GenBank Accession No. NM_001160075.1). The primer sequences for occludin were: forward primer 5-GGGGCTATACAGATCCACGA -3 and reverse primer 5-ATCACCAATGCAGCAATGAA -3 (GenBank Accession No. NM_001163647.2).

### Western blot analysis of tight junctional proteins

Occludin protein expression was analyzed by Western blot. Total protein was extracted in a homogenization buffer (20mM Tris (pH 7.5), 5mM EGTA, 0.1% SDS, 10 μL/mL PMSF, 1% Triton X-100, protease inhibitor cocktail (Calbiochem, CA), and 15mM 2-mercaptoethanol) from 12 vocal fold epithelial samples challenged with 100 μM acrolein or control for 3 hours. A BSA assay kit was used to quantify the protein concentration. Isolated samples were boiled in the 2xLaemmli sample buffer (Bio-Rad) for 5 min. Samples were loaded onto the SDS-polyacrylamide gel and electrophoresed. The proteins were then transferred onto a PVDF membrane from the gel. The PVDF membrane was blocked in the 5% milk for 1 hr and incubated in the primary anti-occludin (1:1000) antibody at 4°C overnight. The membrane was then rinsed with mixture of Tris-Buffered Saline and Tween 20 (TBST) and incubated with the HRP linked secondary antibody (Goat anti-rabbit lgG-HRP, 1:3000) for 1 hr. ECL-Western Blotting Substrate was used for membrane immumo-blots, which was visualized and imaged using the Bio-Rad Molecular Imager. After imaging, the membrane was washed with tripping buffer for 15 min, and rinsed with TBST three times. Next, the membrane was incubated with primary anti- β-actin (1:10000) antibody overnight at 4°C, followed by incubation with the secondary antibody (Goat anti-mouse lgG-HRP, 1:5000) for 1 hr. The image of immune-blots for β-actin was obtained in the same procedure as described above. The intensity of bands for occludin (63 kDa) and β-actin (42 kDa) were quantified using ImageJ software (NIH, Bethesda, Maryland). The relative expression of occludin was normalized to β-actin.

### Cell membrane integrity assessment

The cell membrane integrity was analyzed using a lactate dehydrogenase (LDH) assay. Twelve vocal fold epithelial tissues were punched (6 mm in diameter) and incubated in oxygenated HBSS media at 37°C for 1 hour to fully release the LDH from the damaged cells. The tissues were placed in wells filled with either oxygenated control media or media with 100 μM acrolein. The media were then collected and diluted 10 times prior to assay. To avoid the interference caused by acrolein-protein adducts in the media containing LDH, 3% bovine albumin was added to the diluted media and incubated for 2 hr. The concentration of LDH in the sample media were detected using Pierce LDH Cytotoxicity Assay kit following manufacturer’s direction. An aliquot (50 μL) of diluted sample medium was mixed with 50 μL LDH reaction mixture in each well in a 96 wells plate. The absorbance was measured at 490 nm with the subtraction of absorbance at 680 nm. The absorbance was normalized by the weight of each tissue sample. The percentage of LDH release was normalized by the average of the control group.

### Determination of lipid peroxidation

The product of lipid peroxidation, 4-HNE, was detected using immunohistochemistry. Eight vocal fold epithelial samples (6 mm in diameter) were incubated with or without 100 μM acrolein. The samples were fixed in 4% prophenol aldehyde for 1 week and dehydrated in 30% sucrose for 1 week at 4°C. Tissues were frozen in liquid nitrogen before being sliced in microtome. Tissues were sectioned from apical side to basolateral side in the thickness of 35 μm. The sections were stored in cryoprotectant solution at -20°C (30% sucrose, 30% ethylene glycol, 0.05M PBS) before staining.

Four sections from each vocal fold sample were taken and washed in PBS followed by blocking in 5% normal donkey serum (NDS) with 0.3% Triton X-100, rotating for 90 min. The sections were then treated with primary anti-occludin antibody (1:1000) and anti-4 HNE antibody (1:100) at 4°C, overnight, followed by incubation with Alexa Fluor488 goat anti-rabbit IgG (HþL) antibody (1:500) and cyanine goat anti-mouse IgG (1:500) at room temperature (RT) for 1.5 hr. Sections were rinsed with PBS between incubations of different antibodies, and mounted to slides with ProLong Gold anti-fade reagent and dried at room temperature overnight. Sections were imaged using a confocal microscope (C1-plus, Nikon). Images were analyzed using NIS Elements BR software. Confocal images (2~3 images covering entire epithelial area for each section) were used to quantify the intensities of 4-HNE and occludin in epithelia. The intensity quantification data from 4 sections of each vocal fold sample were collected. In one sample exposed to acrolein, two sections were damaged, leaving only 2 sections for further analysis. The average intensity of each vocal fold sample was calculated and processed to examine differences between control and acrolein exposed groups. Fifteen circles with diameter of 30 μm were applied systematically and covered almost the entire area of the epithelium in each image. The average intensities of each circle and average intensities of all fifteen circles in each image were calculated and shown by the software. The average intensity of the target signal of each section was calculated by using the average intensity of each image. The average intensity of each vocal fold epithelium and the mean of the intensity of all four vocal folds in each group were further calculated based on the average intensity of each section.

### Statistical analysis

All data are presented as mean ± SE. Statistical analyses were conducted using IBM SPSS for Windows (version 20, Chicago, Illinois). Analysis for tissue metabolic activity was performed using one-way ANOVA followed by Dunnett’s multiple comparison tests. Analyses of the differences between control and acrolein-exposed groups for TEER values and permeability were carried out by independent t-tests. All other analyses were completed with paired t- test. Differences between two means were considered significant if p values were equal or less than 0.05.

## Results

### Decreased epithelial metabolic activity following acrolein exposure ex vivo

**Fig 1:**
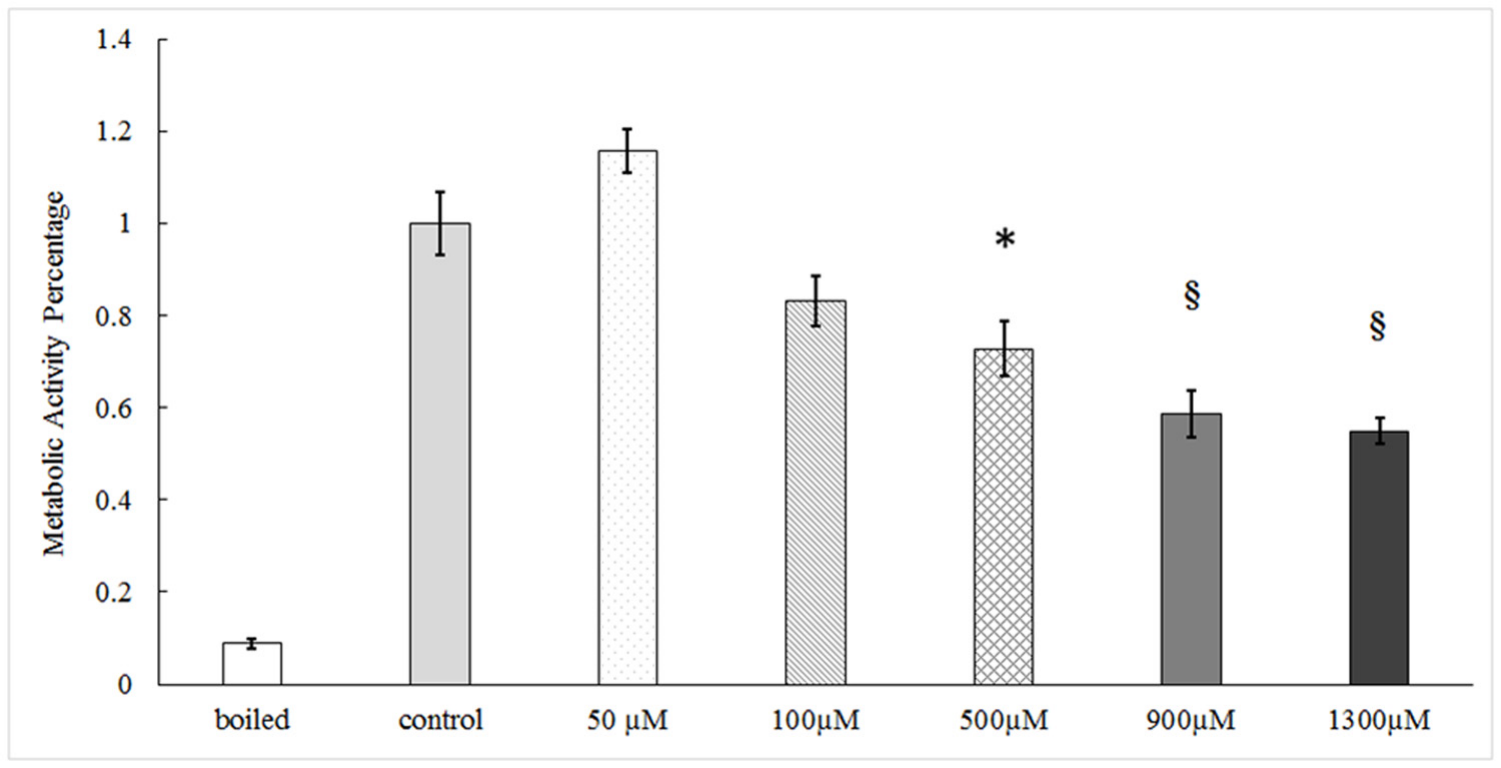
The metabolic activities of vocal fold tissue following acrolein exposure ex vivo. Epithelial metabolic activity was determined by the MTT assay. Tissues were incubated with acrolein as the concentration indicated for 3 hours. Data represent Mean ± SE, n = 7, *: p < 0.05, §: p < 0.001 as compared to controls.

Following incubation with acrolein at the concentration from 50 to 1300 μM, the cell metabolic activity of the vocal fold epithelia showed a dose-dependent decline (Fig 1). The significance became evident when acrolein concentrations equaled or exceeded 500 μM (N = 7, F = 19.413, *p*<0.001). At concentrations of 500 μM, there was a 27.2% (*p*≤0.001) reduction in metabolic activity as compared to controls.

### Impaired barrier function following acrolein exposure

**Fig 2:**
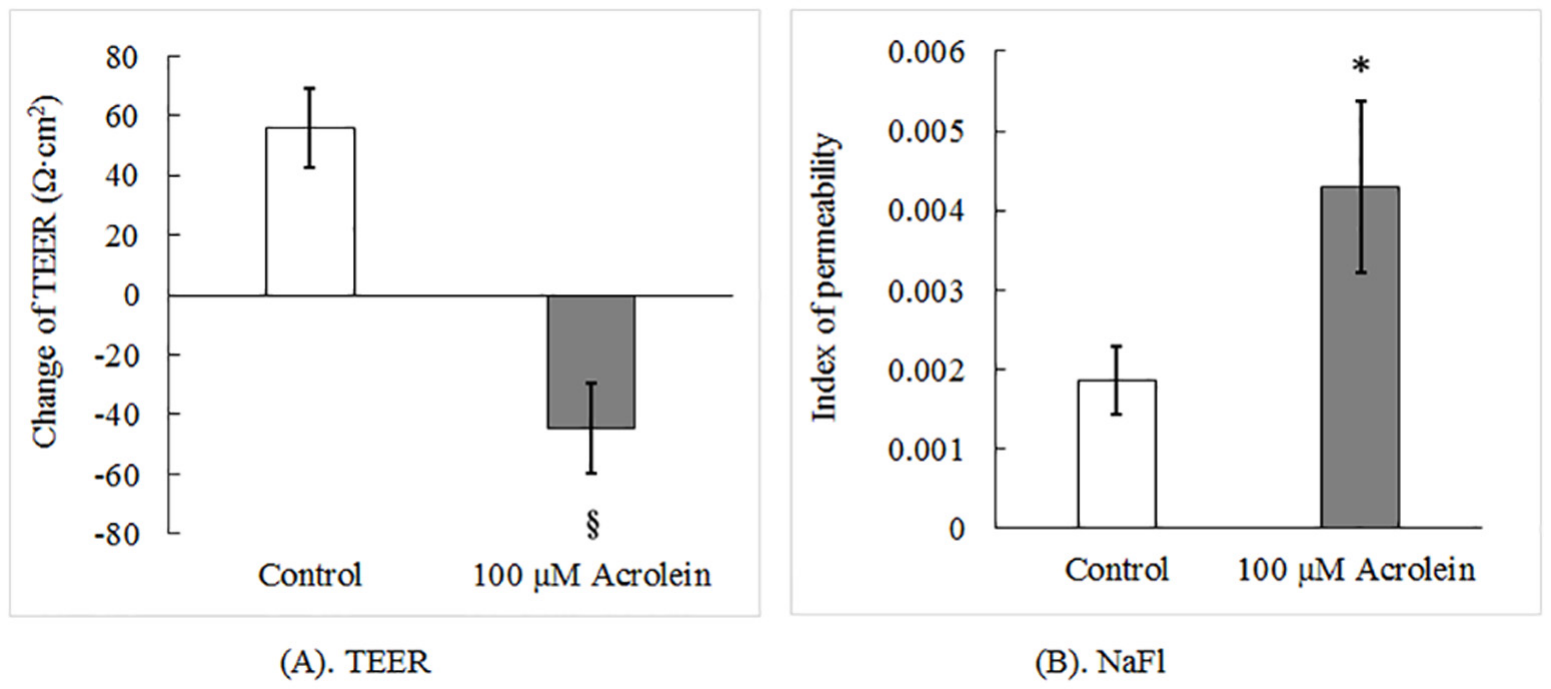
Barrier permeability assay with or without acrolein exposure. Tissues were incubated with acrolein as the concentration indicated for 3 hours. (A). The TEER values were assessed by Ussing chamber system and associated voltage clamp. (B). The epithelial permeability was measured by fluorescent marker NaFI. Index of permeability represents the percentage of fluorescent marker passing though epithelium. Data represent Mean ±SE, n = 7, *: p < 0.05 as compared to controls.

The values of TEER reflect the tightness of the cellular monolayer formed between adjacent cells. In control tissues without acrolein, the TEER values increased by 55.8 Ω·cm^2^ after 3 hr incubation, while the acrolein-treated tissues showed a 44.7 Ω·cm^2^ decrease in the TEER value, a reduction of 180.0% compared to the controls (N = 7, t = 5.023, *p* < 0.001) (Fig 2A), suggesting a reduced tightness of this vocal fold epithelial monolayer.

Further analysis of the barrier integrity by examining the leakage of a large molecular weight compound NaFl demonstrated that the concentration of NaFl in the basolateral chamber was increased by more than 2 fold (N = 7, t = -2.224, *p* = 0.045) (Fig 2B). Thus, both the TEER values and NaFl assay provided unequivocal evidence suggesting a damaged vocal fold epithelial barrier after acrolein exposure.

### Acrolein exposure did not affect the expression of tight junctional proteins

**Fig 3:**
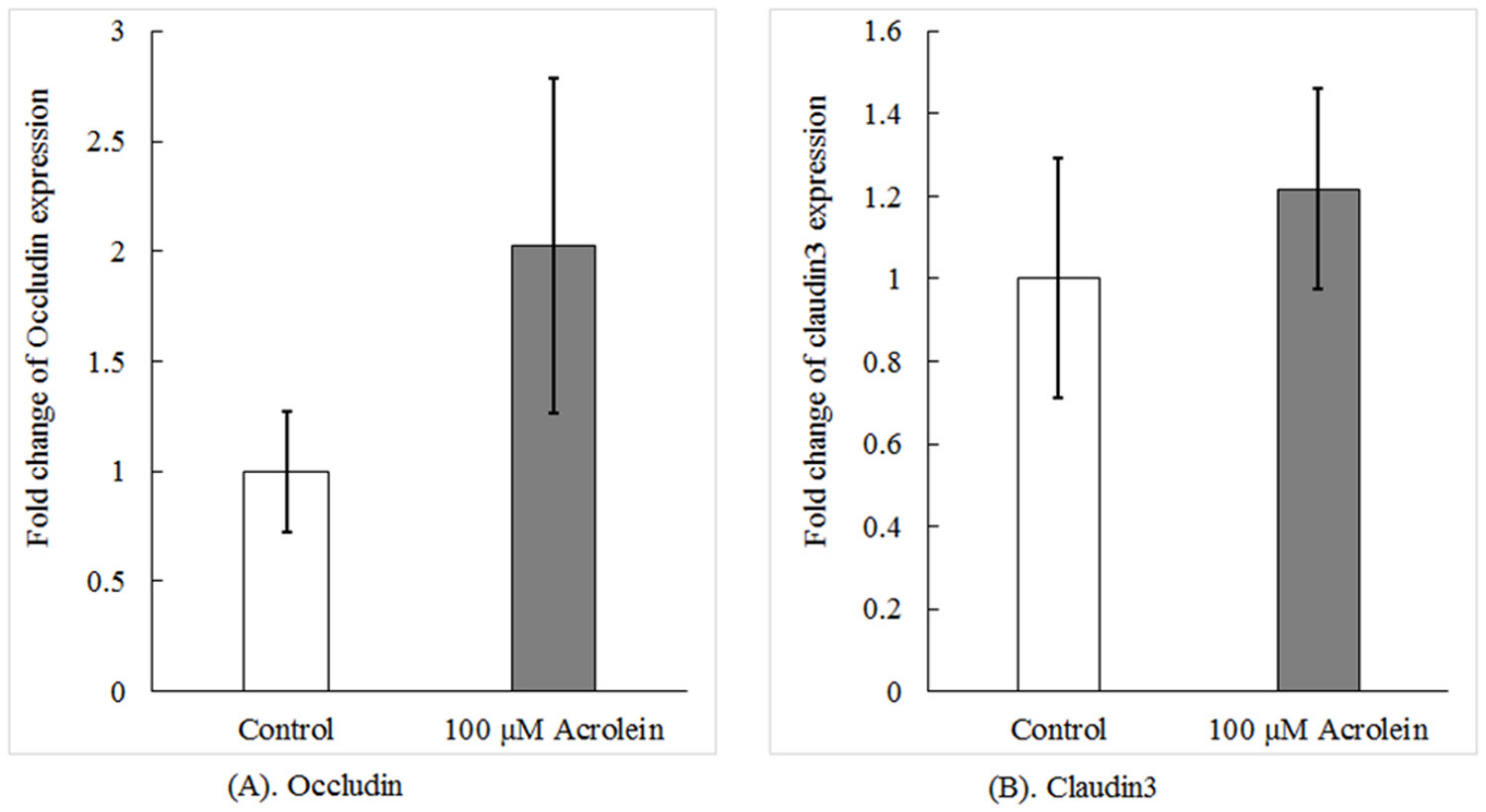
Expression levels of mRNAs encoding typical tight junctional proteins. Tissues were incubated with acrolein as the concentration indicated for 3 hours. The mRNA levels of occludin (A) and claudin3 (B) were determined by qPCR. Data represent Mean ± SE, n = 6 (p = 0.259 for occludin and p = 0.556 for claudin3) as compared to controls.

Maintaining a tight barrier relies partly on the intact expression of tight junctional proteins. Thus, to understand the mechanism whereby acrolein altered the barrier permeability, we set out to examine if acrolein suppressed the expression of two key tight junctional proteins, i.e., occludin and caludin3, in the vocal fold, leading to openness of the barrier. The qPCR data in Fig 3 demonstrated that acrolein exposure did not significantly alter the level of mRNA encoding occludin (N = 6, t = -1.273, *p* = 0.259), nor did it affect the mRNA level of claudin3 (N = 6, t = -0.631, *p* = 0.556).

**Fig 4:**
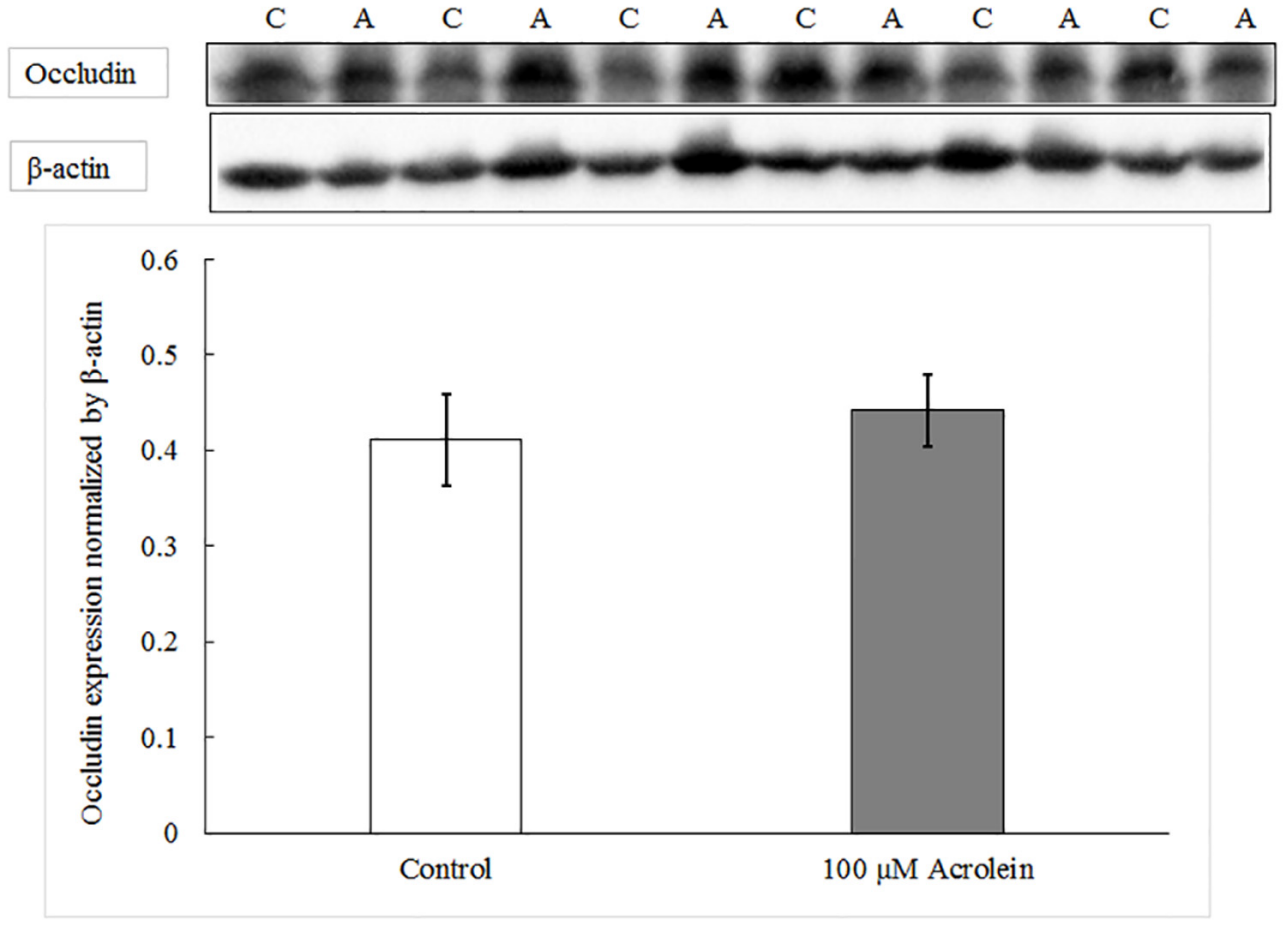
Western blot analysis of occludin protein with or without acrolein exposure. Data represent Mean ± SE, n = 6, p = 0.337 as compared to controls. “C” represents control group and “A” represents acrolein treated group.

To verify the qPCR results, we directly examined the protein expression of occludin. Western blot analysis revealed that acrolein treatment did not affect the protein expression of occludin (data on occludin shown in Fig 4) as compared to controls (N = 6, t = -1.062, *p* = 0.337). Thus, both qPCR and Western blot data established that the effect of acrolein on the tightness of the barrier seemed unlikely to be caused by its action on gene expression of barrier proteins.

### Leakage of cell membrane and increased lipid peroxidation after acrolein exposure

**Fig 5:**
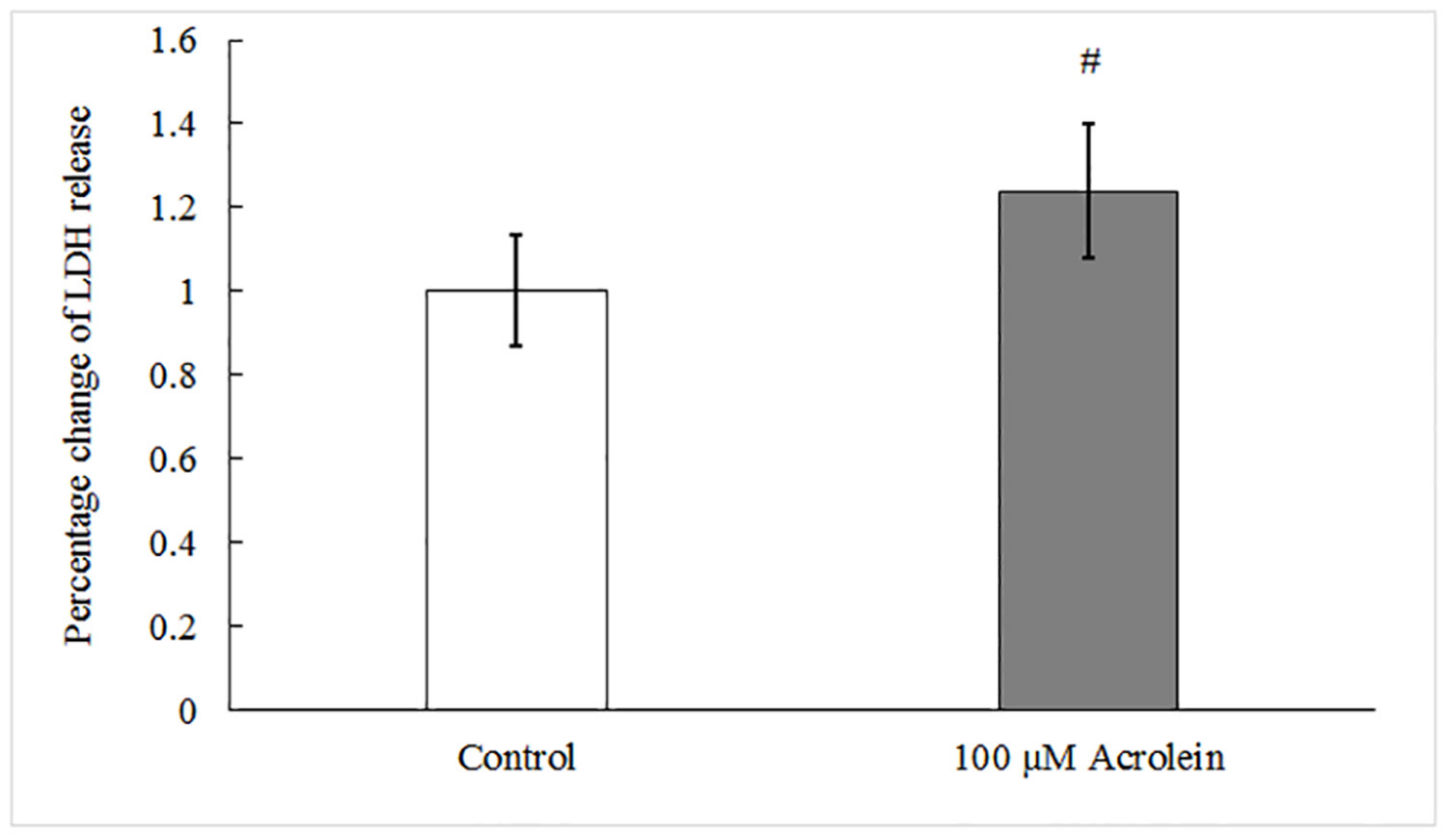
Cell membrane integrity assessed by LDH leakage. Data represents LDH release in extracellular medium normalized by the average in the control group, #: p<0.01 as compared to controls by paired t-test.

LDH is an intracellular protein and usually present inside healthy cells. A significant increase of LDH in the culture medium is an indicator of cell membrane damage. With the assay, there was a 23.7% (N = 6, t = -4.807, *p* = 0.005) increase in LDH activity in the extracellular medium in acrolein-exposed groups as compared to controls (Fig 5). The data suggested that acrolein treatment caused cell membrane damage, which may contribute to acrolein-induced epithelial barrier impairment.

**Fig 6:**
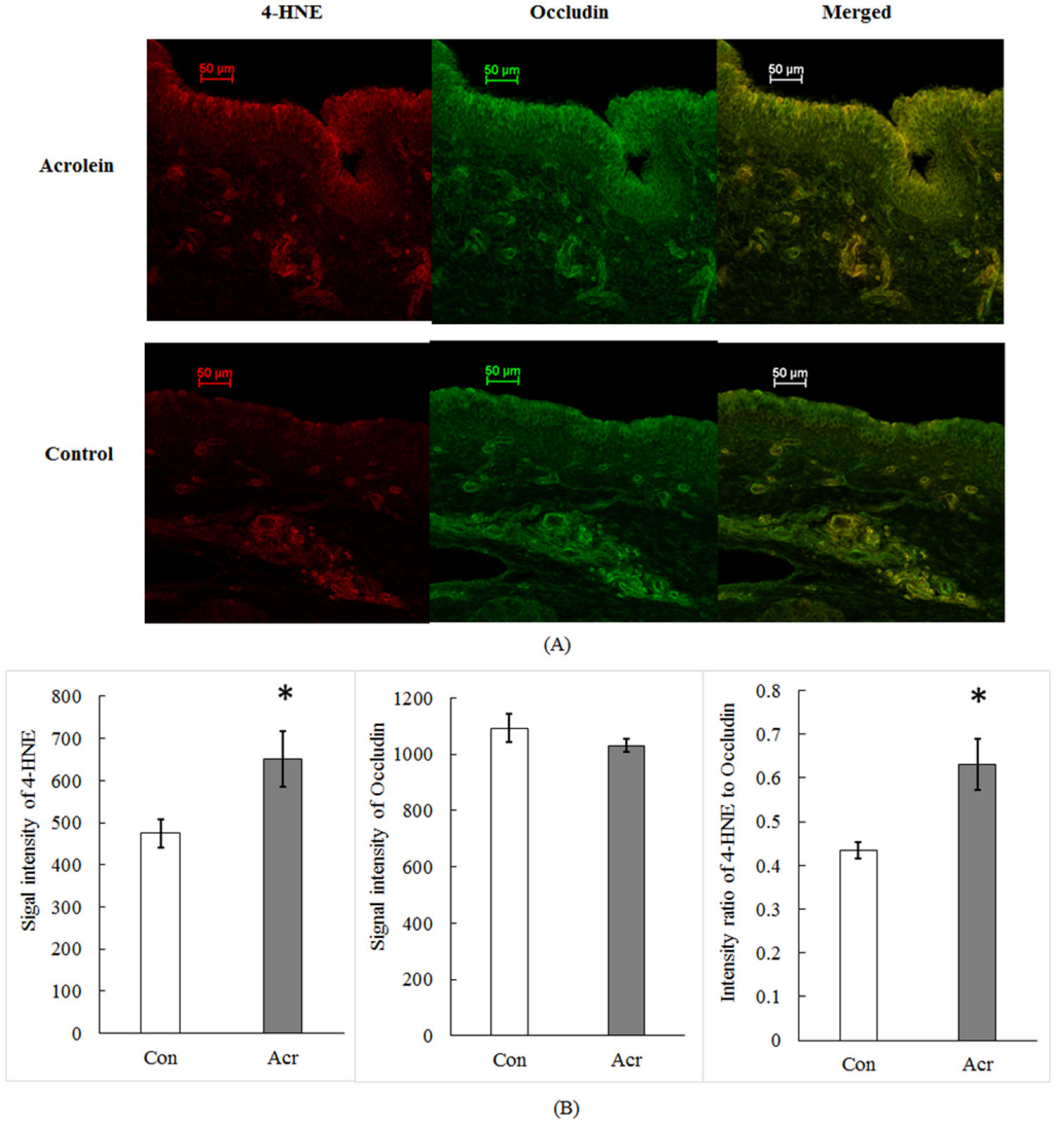
Immunohistochemical study of lipid peroxidation marker with or without acrolein exposure. (A). A typical confocal image of vocal fold epithelia. 4-HNE was stained in red on the left panel; occludin was stained in green on the middle panel; and the merged signals in yellow was present on the right panel. (B). Quantification of signal intensities of 4-HNE and occludin. Data represent Mean ± SE, n = 4, *: p < 0.05 as compared to controls.

The above findings on cell membrane damage led us to question whether acrolein toxicity was associated with the lipid peroxidation, since the latter is often the cause of cell membrane damage. Our confocal data with immunohistochemistry demonstrated that the expression of 4-HNE, a metabolic product of lipid peroxidation, in the vocal fold epithelia was co-localized with occludin (Fig 6A). Acrolein exposure significantly increased 4-HNE fluorescent intensity (N = 4, t = -3.440, *p* = 0.041, Fig 6B) as compared to controls. Further, the ratio of 4-HNE intensity to occludin was greater by 45.6% after acrolein exposure (N = 4, t = -3.767, *p* = 0.033; Fig 6B). These results indicate that the cell membrane damage may be due to the lipid peroxidation caused by acrolein exposure, which underlies acrolein-induced barrier permeability.

## Discussion

The data from this study demonstrate that acrolein exposure disrupted vocal fold epithelial barrier function. The impairment in barrier function occurred at acrolein concentrations that preserved epithelial metabolic activity. The impaired epithelial barrier was due to lipid peroxidation-induced cell membrane damage. This study, for the first time in the literature, reveals that acrolein, a major toxicant of cigarette smoke, may damage vocal fold barrier integrity, leading to inflammation and subsequent voice disorders.

Acrolein is a ubiquitous pollutant in the environment. The concentration in the ambient air in the USA is about 0.5 to 3.186 ppbv [15]. Per recommendations from the National Institute for Occupational Safety and Health (NIOSH) and Occupational Safety and Health Administration (OSHA), the exposure limit to this chemical is 0.1 ppm over an 8 hour time-weighted average (TWA) [38]. However, under some circumstances the concentrations of acrolein are much higher than this recommended level. The concentration of acrolein in cigarette smoke can be as high as 70 ppm [17]. Acrolein concentrations in the smoke after combustion of wood, cotton, and pyrolysis of polyethylene foam range from 50 ppm to 180 ppm (0.41 mg/L) according to various reports [15]. In the current study, we used a 100-μM acrolein solution concentration which is equivalent to 5.6 mg/L. High concentrations have been used in other studies of vocal fold physiology [3] and it is noteworthy that this study involved a very limited exposure duration due to the ex vivo model. Thus, the toxic effects are likely to be even more extensive in vivo albeit at lower doses. This assumption, however, needs further experimental study.

The toxic effect of acrolein has implications for public health. It is estimated that 40.0 million adults (16.8%) in the USA are currently smokers [39]. Besides existing in the ambient environment, acrolein also can be generated endogenously though lipid peroxidation [40] and cancer drug (e.g. cyclophosphamide) metabolism [41]. Lipid peroxidation is a common mechanism in many diseases and pathological conditions. It has also been reported in the vocal fold wound healing literature [42]. Therefore, our study on the effect of acrolein on vocal folds also lays the groundwork for mechanistic investigations of pathological conditions involving lipid peroxidation of the vocal folds.

Upon exposure, acrolein caused a dose-dependent reduction of tissue metabolic activity, and the reduction only became significant when the concentration of acrolein reached at or above 500 μM. When a 100-μM acrolein concentration was chosen for LDH assay, we found significant cell membrane damage. It is possible that cell membrane damage may precede a reduction in metabolic activity following acrolein exposure. The membrane damage could be reversible or irreversible [43]. When the damage is too severe and becomes irreversible, necrosis may occur and metabolic activity will also be reduced. One reason for the different timelines in metabolic activity reduction and cell membrane damage following exposure may be that the cell membrane injury was in the early reversible stage and as such, did not cause mitochondria damage or metabolic dysfunction [43]. The other possible reason may be that the MTT assay is not as sensitive as the LDH assay to acrolein toxicity when cell death is induced by cell membrane injury.

100 μM acrolein adversely affected vocal fold epithelial barrier integrity as measured by a reduction in epithelial resistance and an increase in epithelial permeability. Similar findings have been reported in the literature. Primary culture of monolayer tracheal epithelium showed a reduction in epithelial potential difference and increased permeability following one hour acrolein exposure [44]. Acrolein literature also reports changes in tight junction protein expression in lung epithelia [45]. Acrolein may increase tight junctional protein claudin5 expression at a relatively low dose (30 nM) but has been shown to increase expression at a relatively high dose (300 nM) in human lung endothelial cells following 4-hour exposure [45]. After 24-hour exposure to 10 ppm acrolein, claudin5 expression was upregulated in the mouse lung [45]. Though the effects of acrolein have been quantified on cuboidal airway epithelia, the effects of acrolein exposure on stratified squamous epithelia of the vocal folds has not been investigated. Given the routine mechanical stresses the vocal folds may be exposed to during speaking, it is important to investigate if acrolein exposure has similar effects on the stratified squamous epithelium of the vocal folds.

Xenobiotics can penetrate the epithelium via the paracellular and/or transcellular pathways. In healthy vocal fold epithelium, the tight junction complex seals neighboring epithelial cells by connecting adjacent cell membranes together restricting particle movement. In this study, we did not find any significant change in the expression of occludin and claudin3, two transmembrane proteins that occlude adjacent epithelial cells [46–49] and are expressed in larynx [10, 50]. This indicates that the barrier function reduction is unlikely to be associated with altered gene expression of tight junction proteins. The reason for no change in tight junction protein expression could be attributed to the short exposure duration (3 hours) or a lack of specific effect on the tight junction complex in the vocal folds. It is possible that the functionality of the tight junction proteins may have changed by modifications of protein structure, but this was not investigated in the current study and should be the focus of future work.

Besides the paracellular pathway, epithelial barrier function can also be compromised through the transcellular pathway. Acrolein exposure caused leakage of cell membrane and increased lipid peroxidation. This is expected since acrolein is a highly electrophilic chemical with a great tendency to induce oxidative stress and lipid peroxidation [22, 40, 51]. While the reaction is on the membrane, the consequence is increased barrier permeability, which may allow xenobiotics to gain access to the deep layer of vocal folds. Thus, our data provide new insight into mechanisms of acrolein toxicity on larynx. There are some limitations to the study that must also be discussed. Acrolein concentrations used in the current study were higher than that found in routine environments. Using a high concentration enabled the investigation of changes to a variety of underlying mechanisms that regulate barrier function, and future studies will look at more physiologically relevant levels. A higher concentration was also needed as we were studying an acute exposure rather than documenting the chronic effects. Future studies will also study the effects of removing acrolein via reversal and measuring specific xenobiotic penetration. In vivo studies using environmental chambers will also help us understand the effects of chronic exposure and cumulative effects of acrolein on vocal folds and airway tissue.

## Conclusion

Acute acrolein exposure impairs vocal fold epithelial barrier integrity. The reduced barrier function is associated with lipid peroxidation damage on cell membrane. The damaged barrier may lead to the invasion of xenobiotics to the deep layer of the vocal folds. These data lay the groundwork for a future mechanistic study of inflammation on vocal folds caused by acrolein exposure in vivo.
